# Towards a natural treatment for mania: red onion husk extract modulates neuronal resilience, redox signalling, and glial activation

**DOI:** 10.1186/s40345-024-00338-7

**Published:** 2024-05-09

**Authors:** Chukwuma Raphael Ekeanyanwu, Chidinma Lynda Ekeanyanwu, Kingsley Nnaemeka Ugochukwu

**Affiliations:** Department of Biochemistry, Imo State University, Owerri, Imo State Nigeria

**Keywords:** Red onion husk extract, Mania, Ketamine model, Neurochemical modulation, Mood disorder, Natural compound, FT-IR, GC-MS, Bioactive compounds

## Abstract

**Background:**

Red onion husk, a readily available agricultural waste material, contains diverse bioactive compounds with potential health benefits. This study aimed to assess the safety and therapeutic potential of red onion husk extract in managing manic-like symptoms and associated neurochemical dysfunctions.

**Methods:**

Acute and repeated oral dose studies were conducted in mice and rats to evaluate the safety profile of the extract. FT-IR analysis identified functional groups in the extract, while GC-MS analysis identified specific bioactive compounds in the flavonoid-rich fraction. A ketamine-induced manic behaviour model in Wistar rats was employed to assess the extract’s efficacy in attenuating manic-like symptoms. Behavioural and neurochemical analyses were performed to further investigate the extract’s effects.

**Results:**

The extract demonstrated a favourable safety profile in both acute and repeated dose studies. FT-IR analysis revealed a complex mixture of organic compounds, including hydroxyl groups, alkynes/nitriles, aromatic and non-aromatic C = C bonds, amines, and polysaccharides. GC-MS analysis identified 17 bioactive compounds, including five-methyl-2-phenylindolizine, methadone N-oxide, and 3-phenylthiane, S-oxide. Ketamine administration significantly increased oxidative stress markers, TBARS, and suppressed antioxidant enzyme activities (SOD, GPx, CAT) in both the cerebral cortex and hippocampus, alongside elevated acetylcholinesterase (AchE) activity, indicating enhanced neuronal excitability. Pre-treatment with FRF (25 mg/kg) effectively mitigated ketamine-induced oxidative stress, as evidenced by reduced TBARS levels and partially restored SOD and GPx activities. Interestingly, FRF significantly increased CAT activity (*p* < 0.001), potentially suggesting an additional compensatory mechanism. Notably, FRF pre-treatment also counteracted ketamine-upregulated AchE activity, offering neuroprotection against heightened neuronal excitability.

**Conclusion:**

Red onion husk extract exhibits a favourable safety profile and exerts potent antioxidant and neuroprotective effects, possibly through modulating Nrf2 signalling pathways. Its ability to counteract ketamine-induced oxidative stress and neuronal hyperactivity highlights its potential as a complementary therapeutic strategy for managing manic episodes in bipolar disorder. Further research is warranted to elucidate the precise molecular mechanisms underlying FRF’s action and explore its clinical efficacy in human studies.

## Introduction

Growing interest in the bioactive potential of fruits, vegetables, and herbs has paved the way for investigating their therapeutic applications (Aremu et al. 2019; Hariharapura et al. 2014; Malfa et al. 2020). These naturally occurring plant-based compounds have shown promise in the treatment of bipolar disorder (BD), a complex mental health condition characterized by fluctuations in mood (Grunze et al. 2021). Several studies have explored the potential of phytochemicals to manage BD symptoms and improve patient outcomes. One class of phytochemicals that has garnered attention is omega-3 fatty acids (Grunze et al. 2021). Research has indicated that omega-3 fatty acids, such as docosahexaenoic acid (DHA), may have a beneficial effect on BD. A study found that the omega-3 fatty acid DHA affected the growth of inositol pathway mutants, suggesting a potential mechanism of action similar to that of lithium and valproate, two commonly used mood stabilizers (Ozer et al. 2010). This finding implies that omega-3 fatty acids may modulate the inositol biosynthetic pathway, which has been implicated in the pathophysiology of BD (Ozer et al. 2010). Another group of phytochemicals that have shown promise in BD are herbal and dietary supplements (Oral et al. 2009). A naturalistic study found that 29% of patients with BD used a dietary supplement for at least 7 days, and 20% used a supplement long-term (for at least 50% of days). The most commonly used supplements were fish oil, B vitamins, melatonin, and multivitamins (Oral et al. 2009). While the efficacy of these supplements in BD is still being investigated, the high rate of use highlights the need for healthcare providers to obtain detailed information about all dietary supplements taken by patients with BD (Oral et al. 2009). In addition to omega-3 fatty acids and dietary supplements, other phytochemicals have shown potential in the management of BD. For example, curcumin, the active compound in turmeric, has been found to have neuroprotective and mood-stabilizing effects in animal models of BD (Recart et al. 2021). Similarly, resveratrol, a polyphenol found in grapes and red wine, has demonstrated antidepressant-like effects and the ability to modulate neurotransmitter systems involved in BD (Recart et al. 2021).

The vast amount of waste generated by the fruit and vegetable industry, particularly in onion cultivation, presents a unique opportunity for valorization as a source of potent natural antioxidants (Kupaeva and Kotenkova 2019; Bedrníček et al. 2019). Global onion production exceeds 66 million tons per year, with red, yellow-brown, and white varieties being the most prominent (Ren et al. 2020). Onion peel, rich in bioactive compounds with therapeutic potential, represents an underexploited resource (Shabir et al. 2022). It harbours high levels of total flavonoids, total phenols, quercetin, and its derivatives, contributing to its hypocholesterolemic, anti-asthmatic, and anti-carcinogenic properties (Kumar et al. 2022; Moreno et al. 2006; Hassan et al. 2014). Quercetin, in particular, exhibits potent antioxidant activity (Bonaccorsi et al. 2008). Studies in mice showed that chronic quercetin treatment not only reversed the hyperactive behavior induced by methylphenidate (indicating antimanic-like effects) but also prevented the associated increase in oxidative stress. These findings support previous evidence for quercetin’s potential as an antimanic drug with antioxidant properties. Given ongoing clinical trials on its anti-inflammatory and antioxidant effects, quercetin warrants further investigation as a novel treatment for mania (Kanazawa et al. 2017). Inspired by ethnomedicinal knowledge and research highlighting the neuroprotective potential of onion peels (Kianian et al. 2021); we investigated the efficacy of a red onion husk flavonoid-rich extract in a ketamine-induced mania model in rats. The ketamine-induced mania model is a valuable tool in BD research as it rapidly replicates manic-like symptoms in animals, facilitating the study of potential treatments. Ketamine administration in rodents has been established as a model for mimicking mania-like symptoms of bipolar disorder (BD). This model mimics several key features of manic episodes in BD (Ni et al. 2022). These features may include increased activity, sociability, and vocalizations. Sleep patterns might be disrupted, and the rodents might exhibit hyperactivity, decreased inhibitions, and an elevated mood. Several studies have demonstrated the efficacy of ketamine in alleviating depressive symptoms in BD patients. While the precise neurobiological underpinnings remain elusive, recent findings suggest that chronic lithium treatment reverses ketamine-induced mania-like behaviours in mice. This therapeutic effect appears to be mediated by the PI3K-AKT signaling pathway within the medial prefrontal cortex (mPFC), a brain region known to be structurally and functionally abnormal in BD patients (Ni et al. 2022). Acute and sub-acute oral dose toxicity studies ensured the safety of the extract in mice and rats, paving the way for further investigation of its therapeutic potential.

This study sheds light on the promising potential of red onion husk extract as a natural therapeutic option for managing manic symptoms. The extract’s safety profile and the observed amelioration of behavioural and neurochemical dysfunctions in the ketamine-induced mania model warrant further research to elucidate its mechanisms of action and explore its long-term efficacy and safety in larger animal models. This research contributes to the growing body of evidence on the valorization of fruit and vegetable waste for developing sustainable and cost-effective approaches to managing debilitating neuropsychiatric disorders like BD.

## Materials and methods

### Animals

The protocol for all animal tests conducted in this work was approved by the animal experimentation committee of the Department of Biochemistry at Imo State University in Nigeria and was assigned the ethical number IMSU/EC/01/2023. The National Institute of Health Care Guide for the Care and Use of Laboratory Animals (NIH publication #85–23, amended in 1985) was followed in all procedures involving experimental animals. Every attempt was made to lessen the suffering of the animals and to employ fewer animals overall for the experiment. The investigations employed male and female Wistar rats and mice. Animals from the Veterinary Research Institute, Vom, Jos, Nigeria, were obtained since healthy animals were needed. Commercial carrier trucks were used to move the animals from Vom, Jos to Owerri, Imo State. After being transported in plastic cages for animal housing, the animals were put in secondary containers. To lessen the effects of transit, the animals were first kept in the animal house of the Department of Biochemistry at Imo State University in Owerri, Imo State, for two weeks before being transferred to the testing room. To ensure rigorous experimental control and minimize variation, animals were individually housed under standard colony conditions. These cages provided a specific pathogen-free environment and allowed unrestricted access to food and water throughout the acclimatization period. They were also kept in a 12-hour light/dark cycle (lights on at 6:00 a.m.), with a temperature of 25 to 27 0 C and a relative humidity of 40 to 60%, which was measured with a CEM hydrometer (DT-615, Shenzhen, China).

### Onion husk preparation

Onion husk (*Allium cepa* L.) was collected from the local market within Owerri, Imo State. The Onion husk was characterised by neutral smell and taste, as well as without any signs of mould infection. The husks were washed again with distilled water and left open in a porous tray for 10 min to remove excess water, then kept in a drying oven at 50^0^C for 48 h. The dried husk was grounded using a mixer-grinder for the formation of onion husk powder and stored at -30^0^C in air-tight plastic containers for further use.

### Flavonoid-rich fraction (FRF)

As previously reported by Ekeanyanwu and Njoku (2015), the flavonoid-rich fraction (FRF) of the grounded oven-dried onion husk was produced by solvent-solvent extraction with slight remodelling. Here, 1.65 kg of crushed onion husk was macerated for 72 h at room temperature in 10 L of methanol. A cotton cloth was used to filter the macerate, followed by Whatman filter paper No. 1. Liquid-liquid partitioning of the extract produced the FRF. Chloroform (1 L) was used as a solvent extractor for liquid-liquid extraction. The top layer (methanol residue) was then separated using a 1.5 L liquid-liquid extraction method using ethyl acetate to produce the FRF. After that, the top layer of the ethyl acetate fraction was condensed and used to create the FRF.

### Total flavonoids determination

The calibration curve [0.04, 0.02, 0.0025, and 0.00125 mg/ml in 80% ethanol (v/v)] was made using standard quercetin. 75 µl of 5% sodium nitrite was combined with 0.5 ml of the standard solutions and the test sample at 1 g/ml of the FRF. 500 µl of 1 M sodium hydroxide was added after 6 min and after that 150 µl of a 10% aluminium chloride solution was added and let to stand for an additional 5 min. The blank was filled with the equivalent amount of distilled water in place of the 10% aluminium chloride. At 510 nm, the absorbance of the reagent was measured using a blank. The overall flavonoid content was calculated as Mean ± SEM (*n* = 3) and expressed as mg/g of onion husk extract’s quercetin equivalent using the formula below. The flavonoid content of the FRF was evaluated using the linear regression equation derived from the quercetin standard curve;

$${\rm{Y = 0}}{\rm{.0031X + 0}}{\rm{.0159,}}\,{{\rm{r}}^{\rm{2}}}{\rm{ = 0}}{\rm{.9997}}$$

Where y = Absorbance of each fraction.

X = Concentration of quercetin from the calibration curve.

Flavonoid content = $$\frac{V \times C}{M}$$

C = concentration of quercetin from calibration curve (mg/ml).

V = Volume of extract in ml and.

M = weight of extract in grams.

The mean of the three readings was used and the total flavonoids content was expressed in milligrams/gram of quercetin equivalent (The concentration of total flavonoids in the FRF was 316.39 ± 16.78 mg/g quercetin equivalent).

### Qualitative and quantitative phytochemical analysis

Following established protocols as described by Sofowora 1993; Trease and Evans, 1989, and Ayoola et al. (2008), the onion skin husk powder was phytochemically screened for the presence and makeup of certain phytochemicals.

### GC-MS analysis

The Perkin-Elmer Clarus 680 GC (Perkin-Elmer, Inc., USA) was utilized for the GC-MS analysis of FRF. It was outfitted with an Elite-5ms capillary column that was packed with a fused silica column (30 m in length, 0.25 mm in diameter, and 0.25 μm in thickness). The carrier gas was pure helium gas (99.99%) at a steady flow rate of 1 mL/min. An electron ionization energy approach was used for GC-MS spectral detection, with a high ionization energy of 70 eV (electron volts), 0.2s scan time, and fragments spanning from 40 to 600 m/z. A split ratio of 10:1 was employed with an injection quantity of 1µL, and a constant injector temperature of 250^0^C was maintained. The temperature of the column was even for 30 min at 500 C, then increased at a rate of 100 C per minute to 280^0^C. Finally, the temperature was elevated to 300^0^C for 10 min. By comparing the test sample’s retention time (min), peak values, peak height, and mass spectral patterns with those of authentic compounds kept in the National Institute of Standards and Technology (NIST) library, the content of bioactive compounds was found. The names, component composition, and molecular weight of the inquiry materials were found.

### FT-IR spectroscopy analysis

The FT-IR analysis was carried out using the Cary 630 FT-IR Spectrometer (Agilent Technologies Inc., Santa Clara, CA, USA). Using a high-pressure vacuum pump and Whatman No. 1 filter paper, the FRF was sifted via FT-IR analysis after being centrifuged for 10 min at 3000 rpm. The test sample was diluted to a ratio of 1:10 using the same solvent. A Cary 630 FT-IR Spectrometer operating in the 4000–650 nm wavelength range was used to analyze the extract. The peaks were found and their values were recorded.

### Single oral dose toxicity study

An acute oral toxicity study of the FRF was performed using mice according to the Organisation for Economic Cooperation and Development (OECD) guideline 423 (OECD, 2001). Single-dose studies often utilize mice because they require a smaller amount of test compound compared to larger animals. Nine mice were randomly divided into 3 groups of 3 mice each. The FRF and distilled water were orally administered to the mice after overnight fasting at a volume of 10 ml/kg body weight (Ekeanyanwu and Njoku 2014). The mice in group I were administered 300 mg/kg body weight of the FRF dissolved in distilled water. The mice were observed for general behaviour changes; symptoms of toxicity and mortality after treatment for the first 4 h, then over 48 h. Group 2 was administered sequentially at 48-hour intervals with the next higher dose of 2000 mg/kg body weight of the FRF in distilled water when there were no signs of toxicity or mortality showed in group 1 after 48 h of treatment. In parallel, group 3 mice were treated with vehicle (distilled water) to establish a comparative negative control group. All animals were observed at least once during the first 30 min in the first 4 h following vehicle or FRF administration and then once a day for 14 days. According to OECD recommendations (OECD, 2001), this observation was made to determine when clinical or toxicological signs started to appear. Every observation, including alterations to the eyes, mucous membranes, skin, and fur, as well as behavioural tendencies, was methodically documented and kept up to date with a personal file. Observations of convulsions, tremors, diarrhoea, salivation, lethargy, sleep, coma, and mortality were also taken into consideration. The mice in each group were decapitated using a rodent guillotine (Harvard Apparatus, USA) after the study. Their kidney, liver, and heart were promptly removed, and they were then washed in ice-cold saline (0.9% NaCl). Following acute oral toxicity testing, samples from the heart, kidney, and liver were examined histopathologically.

### 14-Day repeated oral dose toxicity study

For a 14-day repeat-dose toxicity study, healthy male Wistar rats were randomly assigned to four groups (5/sex/group). Vehicle (distilled water) or graded doses of the FRF (500, 1000 and 2000 mg/kg of body weight) were administered to rats by oral gavage once daily for 14 days at a dose of 10 ml/kg of body weight. The food composition and water intake are recorded daily. The body weights of animals were recorded shortly before the administration of the tested substance and at the end of each week. The percentage of body weight change is calculated according to the following equation:

$$\eqalign{& {\rm{Percentage}}\,{\rm{body}}\,{\rm{weight}}\,{\rm{change}}\, = \cr & {{Body\,weight\,at\,the\,end\,of\,each\,week\, - \,Initial\,body\,weight} \over {Initial\,body\,weight}}\, \times \,100 \cr}$$

On the 15th day, about 5 ml of blood was collected from the retro-orbital plexus without the use of topical anaesthesia after overnight fasting and sera were prepared from 4 ml of the collected blood by centrifugation at 640 g for 10 min and then stored at 4 °C for different biochemical parameters (Urea, Creatinine, Albumin, Globulin, Total bilirubin, Conjugated bilirubin, Alkaline phosphatase, Alanine transaminase and Aspartate aminotransferase). Analysis of haematological markers, including haemoglobin, white blood cells, neutrophils, lymphocytes, monocytes, Eosinophils, and basophils, was done on the remaining uncoagulated blood from the 5 ml of blood that was collected. Following the collection of blood samples from the rats, the hearts, livers, and kidneys of each group of rats were promptly dissected out and washed in ice-cold saline (0.9% NaCl) after the rats were killed by beheading with a rodent guillotine (Harvard apparatus, USA). The liver, kidney, and heart were removed, fat was removed, and the organs were promptly weighed and blotted with clean tissue paper. Using the following equation, the relative organ weight (ROW) was determined and noted about the body weight:

$$ROW\, = \,{{Absolute\,organ\,weight} \over {\;body\,weight\,at\,the\,time\,of\,sacrifice}}\,{\rm{ \times }}\,100$$

Samples from the vital organs (liver, kidney and heart) of acute oral toxicity tests were subjected to histopathological evaluation. They were fixed in 10% buffered formalin, routinely processed and embedded in paraffin wax. Paraffin Sect. (5 μm) were cut on glass slides and stained with haematoxylin and eosin. An experienced pathologist who was unaware of the experimental groups to which each section belonged conducted the analysis. The slides were examined under a light microscope as earlier stated by Ekeanyanwu and Njoku (2014).

### Animal model of bipolar mania and extract and/or lithium administration

Considering the rigorous nature of the investigative procedures and the need for ample blood and brain tissue samples for analysis, Wistar rats were chosen as the animal model for this study. Thirty animals were divided into six groups of 5 rats each:

- Group I (Normal saline, vehicle),
- Group II (Ketamine, 25 mg/kg),
- Group III (Ketamine 25 mg/kg + Lithium 45 mg/kg),
- Group IV (Ketamine 25 mg/kg + FRF 500 mg/kg),
- Group V (Ketamine 25 mg/kg + Lithium 45 mg/kg + FRF 500 mg/kg),
- Group VI (Ketamine 25 mg/kg + Lithium 22.5 mg/kg + FRF 500 mg/kg).

The animals in Groups IV, V, and VI were given an oral dose of FRF, whereas the animals in Groups III, V, and VI were given Lithium (twice daily). From the eighth to the fourteenth day, the animals in Groups I and II received the same volume of saline solution (10 ml/kg b.wt) orally. Animals in Groups II, III, IV, V, and VI were also given ketamine, while those in Group I were given a vehicle intraperitoneally. The animals were given a single injection of ketamine or saline on the 15th day of treatment. The Locomotor activity was assessed in an open field apparatus thirty minutes later. The FRF, lithium chloride, and ketamine dosages and treatment times were determined using previous research (Spohr et al. 2019) and the findings of the current study.

#### Open field test

An open-field device was used to measure locomotor activity (Gazal et al. 2015; Debom et al. 2016). We applied the model that Wang et al. (2017) described to the animals. A thorough explanation of the improved method was previously given by Ekeanyanwu et al. (2021). Briefly, the study uses an open field box, which is a rectangular space with a hard floor that is 60 cm by 60 cm by 40 cm and is composed of painted white wood. Permanent read markers were used at the bottom to divide the floor into 16 equal squares. Every rat was put in a different part of the field, and we timed how much it moved overall every ten minutes. After the assay, 70% alcohol was used to clean the area and allow it to dry completely before adding a new rat to eliminate olfactory bias.

#### Brain sample preparation and biochemical analyses

##### Brain samples

Following behavioural assessments, rats were euthanized by decapitation with a rodent guillotine (Harvard Apparatus, USA) and the cerebral cortex and hippocampus were dissected on ice. Samples were immediately frozen at -80 °C until further processing.

##### Homogenization

Frozen brain regions were thawed on ice and homogenized in ice-cold KClKH_2_PO_4_ buffer (12 mM KCl, 0.038 mM KH2PO4, pH 7.4) using a Polytron PT 3100 D homogenizer (Thomas Scientific, Thomas Scientific). Homogenates were centrifuged at 10,000 g for 10 min at 4 °C, and the supernatants were collected for subsequent analyses.

##### Thiobarbituric acid reactive species (TBARS) levels

Lipid peroxidation was assessed by measuring TBARS using a modified version of the Wilbur et al. (1949) method. Protein precipitation was performed with 10% trichloroacetic acid (TCA), and the supernatants were reacted with thiobarbituric acid (TBA) reagent at 95 °C for 20 min. Absorbance at 532 nm was measured in a spectrophotometer, and TBARS levels were calculated as nanomoles of malondialdehyde (MDA) equivalents per milligram of protein using a standard curve.

##### Superoxide dismutase (SOD) activity

SOD activity was determined based on its ability to inhibit pyrogallol autoxidation, as described by Misra and Fridovich (1977) with modifications. The reaction mixture containing pyrogallol, carbonate buffer, and homogenate was initiated with hydrogen peroxide (H_2_O_2_). The decrease in absorbance at 420 nm due to inhibition of autoxidation was monitored spectrophotometrically. SOD activity was expressed in units per milligram of protein based on the amount of enzyme required for 50% inhibition and a standard curve.

##### Catalase (CAT) activity

The enzymatic decomposition of H_2_O_2_ was measured to assess CAT activity using the method of Aebi (1984). The reaction mixture containing phosphate buffer, homogenate, and H_2_O_2_ was monitored for oxygen evolution by measuring the change in absorbance at 240 nm over time. CAT activity was expressed in units per milligram of protein based on the initial rate of change in absorbance.

##### Glutathione peroxidase (GPx) activity

GPx activity was determined according to Pagha and Valentine (1967) with modifications. The reaction mixture containing reduced glutathione (GSH), sodium azide, NADPH, glutathione reductase, and homogenate was initiated with H_2_O_2_. The decrease in absorbance at 340 nm due to NADPH oxidation was monitored spectrophotometrically. GPx activity was expressed in units per milligram of protein based on the rate of NADPH oxidation and a standard curve.

##### Acetylcholinesterase (AChE) activity

AChE activity was measured using the Ellman et al. (1961) method with modifications. The reaction mixture containing homogenate, 5, 5’-dithiobis (2-nitrobenzoic acid) (DTNB), and acetylthiocholine iodide was monitored for the formation of the yellow 5-thio-2-nitrobenzoate anion at 412 nm. AChE activity was expressed in units per milligram of protein based on the initial rate of change in absorbance.

### Statistical analysis

Data were reported as mean ± SEM, with *p* < 0.05 considered significant. Group differences were assessed by one-way ANOVA followed by Bonferroni post-hoc tests where appropriate. Interactions between control groups (saline, ketamine) and treatment groups (ketamine + lithium, ketamine + FRF, combined regimens) were evaluated using one-way ANOVA with Bonferroni post-hoc for all measured outcomes (*p* < 0.001).

## Results

### Chemical characterization of onion husk extract

#### The phytochemical analysis of onion husk powder

A phytochemical analysis of onion husk powder revealed the presence of alkaloids (12.35 ± 1.51 mg/kg), cardiac glycosides (3.37 ± 0.36%), flavonoids (18.13 ± 1.01 mg/kg), with tannins being the most abundant at 45.12 ± 2.49%. Onion skin is a good source of a variety of bioactive compounds.

#### FT-IR Chromatogram of FRF of onion husk extract

Based on peak values in the infra-red region, FT-IR spectroscopy (Grube et al. 2008) is a dependable and practical method for detecting various bonds/stretches and phytochemical functional groups. The FRF of onion husk extract was analysed using FT-IR in the study. The peak ratio was used to distinguish between the functional classes of biomolecular components. The FT-IR peak values, intensity and functional groups of FRF of Onion husk extract are presented in Table 1. The FT-IR peak values, intensity, and functional groups are shown in Table 2. There are ten (10) major peaks between 1000 cm^− 1^ and 3300 cm^− 1^. Each band addresses an overall overlap of specific functional group absorption peaks in the test sample. Every absorption spectrum of different components exhibited significant overlap in the spectra. The presence of a strong O-H stretching vibration at 3254.0 cm^-1 indicates the presence of hydroxyl groups in the extract. This suggests the presence of alcohols, phenols, or carboxylic acids. The intense C ≡ C stretching vibration at 2120.9 cm^− 1^ suggests the presence of alkynes or nitriles in the extract. The C = C stretching vibration at 1602.8 cm^− 1^ could be due to aromatic or non-aromatic compounds. Further analysis of the spectrum is needed to determine the specific type of C = C bond. The presence of a peak at 1509.6 cm^− 1^ suggests either aromatic C = C stretching or N-O stretching due to nitro compounds. The C-H bending vibration at 1446.2 cm^− 1^ indicates the presence of alkanes, alkenes, or alkynes in the extract. The C-N stretching vibration at 1379.1 cm^− 1^ suggests the presence of amines. The C-O stretching vibrations at 1252.4 and 1200.2 cm^− 1^ indicate the presence of esters, ethers, alcohols, and aromatic compounds. The peak at 1166.7 cm^− 1^ is likely due to C-C stretching vibrations. The peak at 1036.2 cm^− 1^ suggests the presence of C-O stretching vibrations in polysaccharides. The described infrared functional group characteristics were reported in the literature (Nandiyanto 2019).

**Table 1:** FT-IR peak values, intensity and functional groups of FRF of onion husk extract

S/*N*	Frequency (cm^− 1^)	Intensity (%)	Assignment
1	3254.0	44.060	OH stretching (alcohols, phenols, carboxylic acids)
2	2120.9	94.429	C ≡ C stretching (alkynes, nitriles)
3	1602.8	46.251	C = C stretching (aromatic or non-aromatic)
4	1509.6	58.323	Aromatic C = C stretching or N-O stretching (nitro compounds)
5	1446.2	55.528	C-H bending (alkanes, alkenes, alkynes)
6	1379.1	51.525	C-N stretching (amines)
7	1252.4	43.137	C-O stretching (esters, ethers, alcohols)
8	1200.2	42.826	Aromatic C-O stretching
9	1166.7	42.269	C-C stretching
10	1036.2	32.002	C-O stretching (polysaccharides)

### GC-MS chromatogram of FRF of onion husk extract

GC-MS chromatogram of FRF of Onion husk extract showed 17 peaks which indicated the presence of 17 different bioactive/phytochemical compounds (Table 3). The results revealed that the percentage of major bioactive compounds viz., Hexadecane − (0.1194%), [2.2]Paracyclophane − (0.7093%), 3-Benzyl-5.chloro-1,2,3-triazole-1-oxide − (1.1421%), Benzene, 1,1’-(1,2-cyclobutanediyl)bis-, trans-(8.8655%), Octadecane − (0.0962%), Methyl 2,4,6-trihydroxybenzoate – (0.2404%), n-Hexadecanoic acid – (2.8762%), 5-Eicosene, (E) – (1.5377%), Octadecyl propyl ether – (1.1582%), Carbonic acid, allyl tetradecyl ester – (0.2882%), 8-Methyl-6-nonenamide – (1.8032%), 3-Phenylthiane, S-Oxide (10.6738%), 1-Cyclohexylnonene − (0.7823%), Methyl hexadecyl ether − (0.475%), Methadone N-oxide − (21.9151%), 5-Methyl-2-phenylindolizine − (46.9458%), and (1-Ethylbuta-1,3-dienyl)benzene − (0.3716%) were found as the major compounds in the FRF.

**Table 2:** Bioactive compounds present in the FRF of red onion (*Allium cepa* L.) husk extract

Peak	Name of the compound	Retention time (min)	Peak area (%)	Quality	Molecular formula
1.	Hexadecane	13.3589	0.1194	91	C_16_H_34_
2.	[2.2]Paracyclophane	14.5667	0.7093	91	C1_6_H_16_
3.	3-Benzyl-5.chloro-1,2,3-triazole-1-oxide	15.4432	1.1421	35	C_9_H_8_ClN_30_
4.	Benzene, 1,1’-(1,2-cyclobutanediyl)bis-,trans-	15.9866	8.8655	91	C_16_H_16_
5.	Octadecane	17.7204	0.0962	83	CH_3_(CH_2_)_16_CH_3_
6.	Methyl 2,4,6-trihydroxybenzoate	19.4577	0.2404	93	C_8_H_8_0_5_
7.	n-Hexadecanoic acid	21.7238	2.8762	99	C_16_H_32_O
8.	5-Eicosene, (E)-	24.8828	1.5377	68	C_20_H_40_
9.	Octadecyl propyl ether	25.3959	1.1582	49	C_21_H_44_0
10.	Carbonic acid, allyl tetradecyl ester	26.7633	0.2882	20	C_18_H_34_0_3_
11.	8-Methyl-6-nonenamide	28.2009	1.8032	41	C_10_H_19_NO
12.	3-Phenylthiane, S-Oxide	28.7745	10.6738	27	C_11_H_14_OS
13.	1-Cyclohexylnonene	29.3672	0.7823	70	C_15_H_28_
14.	Methyl hexadecyl ether	29.4778	0.475	56	C_17_H_36_O
15.	Methadone N-oxide	30.0923	21.9151	59	C_21_H_27_NO
16.	5-Methyl-2-phenylindolizine	30.3514	46.9458	46	C_15_H_13_N
17.	(1-Ethylbuta-1,3-dienyl)benzene	32.1491	0.3716	22	C_11_H_12_

Onion husks, a byproduct often discarded, were found to be rich in various health-promoting compounds. Analysis revealed the presence of alkaloids, flavonoids, and other bioactive compounds with tannins being the most abundant. Further investigation using FT-IR confirmed the presence of functional groups indicative of phenols, alcohols and carboxylic acids. Finally, GC-MS identified 17 distinct bioactive compounds with Methadone N-oxide and 5-methyl-2-phenylindolizine being the most prominent. These findings suggest that onion husks have the potential to be a source of valuable bioactive compounds.

### Toxicological profile of FRF of red onion (*Allium cepa* L.) husk extract

**Table 3:** Body weight (g) values of wistar rats orally administered with FRF of onion husk extract in a single-dose toxicity study

Week	Group I (300 mg/kg b.wt.)	Group II (2000 mg/kg b.wt.)	Group III (Distilled water
Week 1	114.16 ± 8.72^b^	110.37 ± 2.48^a, b^	121.07 ± 7.55^a, c^
Week 2	139.58 ± 8.10	140.88 ± 2.37^a^	147.50 ± 1.09^a, b^
Week 3	156.35 ± 7.02^b^	149.70 ± 3.24^b^	162.11 ± 4.50^b, c^

Data are expressed as mean ± SEM. Mean values with the same superscript letters are significantly different at *p* < 0.05

**Table 4:** Body weight values of wistar rats orally administered with FRF of onion husk extract in a 14-day repeat dose toxicity study

Week	Group I (Distilled water)	Group II (500 mg/kg b.wt.)	Group III (1000 mg/kg b.wt.)	Group IV (2000 mg/kg b.wt.)
Week 1	107.34 ± 3.35^a, c^	104.64 ± 5.69^a, b^	114.79 ± 4.08^a, b^	115.69 ± 3.27^a, b^
Week 2	134.82 ± 4.36^a, b^	135.98 ± 6.34^a^	139.20 ± 6.58^a, c^	132.73 ± 4.97^a, c^
Week 3	147.86 ± 3.56^b, c^	145.98 ± 5.90^b^	144.08 ± 4.58^b, c^	144.27 ± 3.90^b, c^

Data are expressed as mean ± SEM. Mean values with the same superscript letters are significantly different at *p* < 0.05

**Table 5:** Relative organ weight (mg/g) of wistar rats orally administered with FRF of Onion husk extract after a 14-day repeat dose toxicity study

Group	Liver	Kidney	Heart
Group I (Distilled water)	4.21 ± 0.32	0.38 ± 0.02	0.42 ± 0.01
Group II (500 mg/kg b.wt.)	4.26 ± 0.15	0.33 ± 0.03	0.44 ± 0.04
Group III (1000 mg/kg b.wt.)	3.80 ± 0.12	0.31 ± 0.01	0.42 ± 0.03
Group IV (2000 mg/kg b.wt.)	4.34 ± 0.29	0.39 ± 0.02	0.44 ± 0.01

Data are expressed as mean ± SEM. Mean values with the same superscript letters are significantly different at *p* < 0.05

**Fig. 1:**
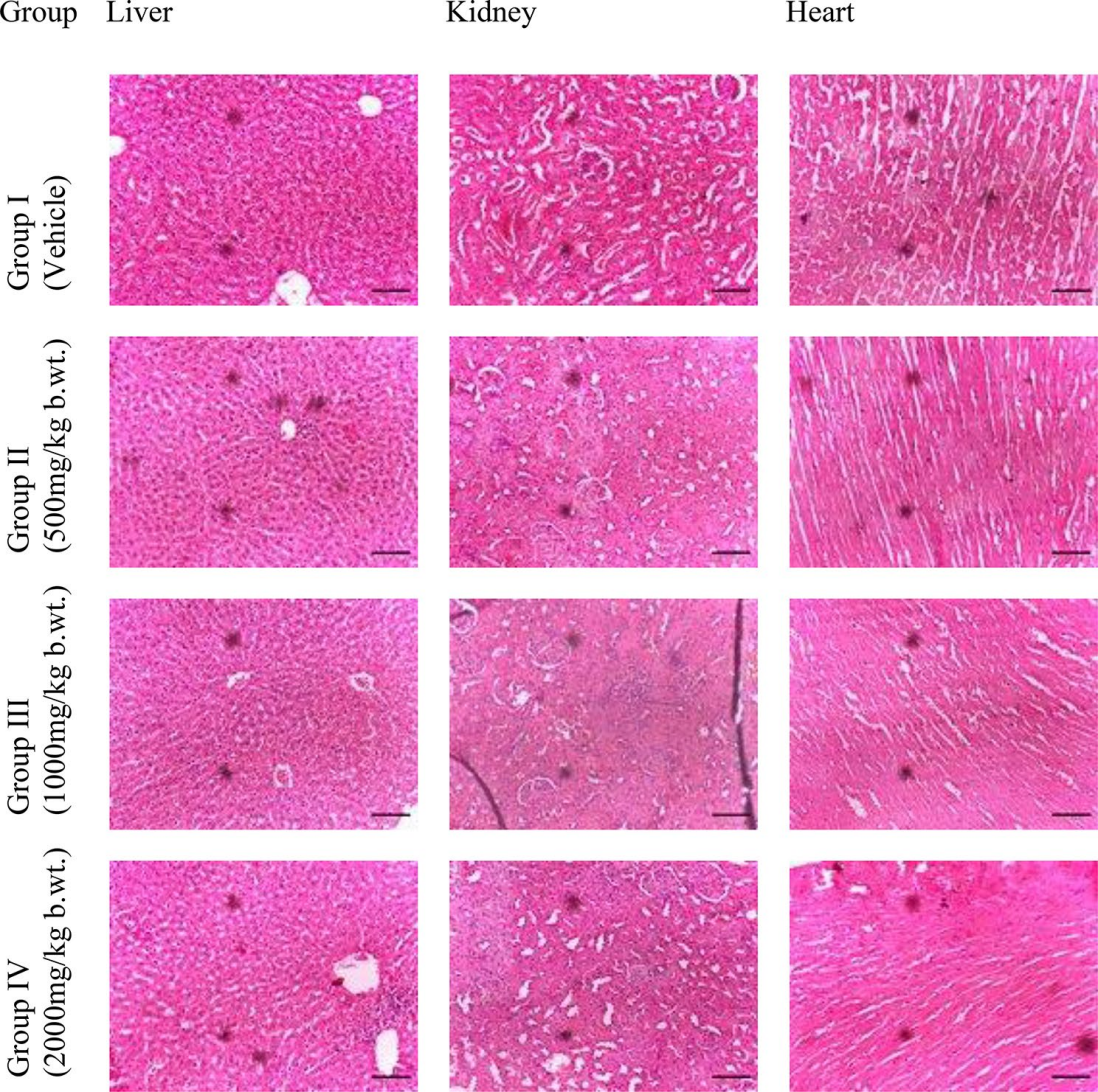
Histology of the liver, kidney and heart of Wistar rats orally administered with FRF of onion husk extract in a 14-day repeat dose toxicity study (400 ×)

#### Body weight

The provided results suggest that oral administration of the FRF did not affect the body weight of rats in both single-dose and 14-day repeated-dose toxicity studies. This indicates that the extract, at the tested doses, did not induce weight gain or loss, suggesting no significant influence on the animals’ overall metabolism or energy balance (Tables 3 and 4).

#### Organ weight and relative organ weight

Similar to body weight, no significant changes in the weights of liver, kidney, and heart were observed between the control and treatment groups after 14 days of oral administration (Table 5). This further supports the conclusion that the FRF, at the studied doses and duration, did not pose any adverse effects on vital organs in the tested animals.

#### Biochemical and haematological parameters

Table 6 shows the effect of FRF of Onion husk on biochemical and haematological parameters in rats. After administering FRF of Onion husk extract for 14 days, Wistar rats displayed decreased creatinine levels, increased total protein in groups 2 and 3, decreased globulin in groups 3 and 4, and increased bicarbonate in groups 3 and 4 compared to the control group. Additionally, all treated groups showed decreased alkaline phosphatase levels compared to the control group. Alanine transaminase levels decreased in groups 2 and 4, while aspartate aminotransferase levels increased in groups 3 and 4 compared to the control. Dose-dependent changes occurred in total protein (increasing), globulin (decreasing), bicarbonate (increasing), alkaline phosphatase (decreasing), and aspartate aminotransferase (increasing). Overall, FRF of Onion husk extract was well-tolerated at doses of 500 and 1000 mg/kg b.wt, but higher doses may cause adverse effects like increased aspartate aminotransferase levels. After 14 days of treatment, there was no significant (*P* > 0.05) effect on total white blood cells in the treated group compared to the control group. There was no significant (*P* > 0.05) effect on the lymphocyte count in the treated group administered 2000 mg/kg b.wt FRF of Onion husk compared to the control group. However, significant differences (*P* < 0.05) were observed in the Monocytes and Granulocytes count in the treated group administered 2000 mg/kg b.wt the FRF of Onion husk compared to the control group administered distilled water only. Red blood cells, haemoglobin, erythrocyte sedimentation rats, and packed cell volume were not significantly different (*P* > 0.05) in the test groups compared to the control group. There was also no significant (*P* > 0.05) effect on mean corpuscular volume, mean corpuscular haemoglobin, and mean corpuscular haemoglobin concentration in the treated groups compared with the control group.

### Histopathological changes

Histopathological examinations were performed on the liver, kidneys and heart to assess possible organ damage (Fig. 1). The normal rat liver has a microscopic architecture structured in hexagonal lobules and acini. The hexagonal lobules are centred on the central vein, with surrounding hepatocyte cords and have a portal triad containing branches of the hepatic artery, the bile duct, and the portal vein. The photomicrograph of the liver section of the normal rats shows a normal central vein, lamella of hepatocytes and sinusoids. Other stromal elements appear normal. Adverse effects were not found in the liver of rats administered 500 mg/kg b.wt. and 1000 mg/kg b.wt of FRF of Onion husk orally. The kidneys of normal rats showed normal glomeruli, Bowman’s capsule, tuft, tubules and stromal cells. Adverse effects were not found in the kidneys of rats administered 500 mg/kg and 1000 mg/kg b.wt. FRF, however, the histological section of the kidney of rats shows areas of haemorrhage, congested tubules and normal glomeruli with a slight increase in Bowman’s capsule space. The histological section of the heart tissues of normal rats shows unremarkable cardiac muscle cells within an intact tissue stroma. Adverse effect was not found in rats administered 500 mg/kg b.wt, 1000 mg/kg b.wt and 200 mg/kg b.wt FRF of Onion husk extract.

### Effect of FRF of red onion (Allium cepa L.) husk extract on manic-like behaviour

**Fig. 2:**
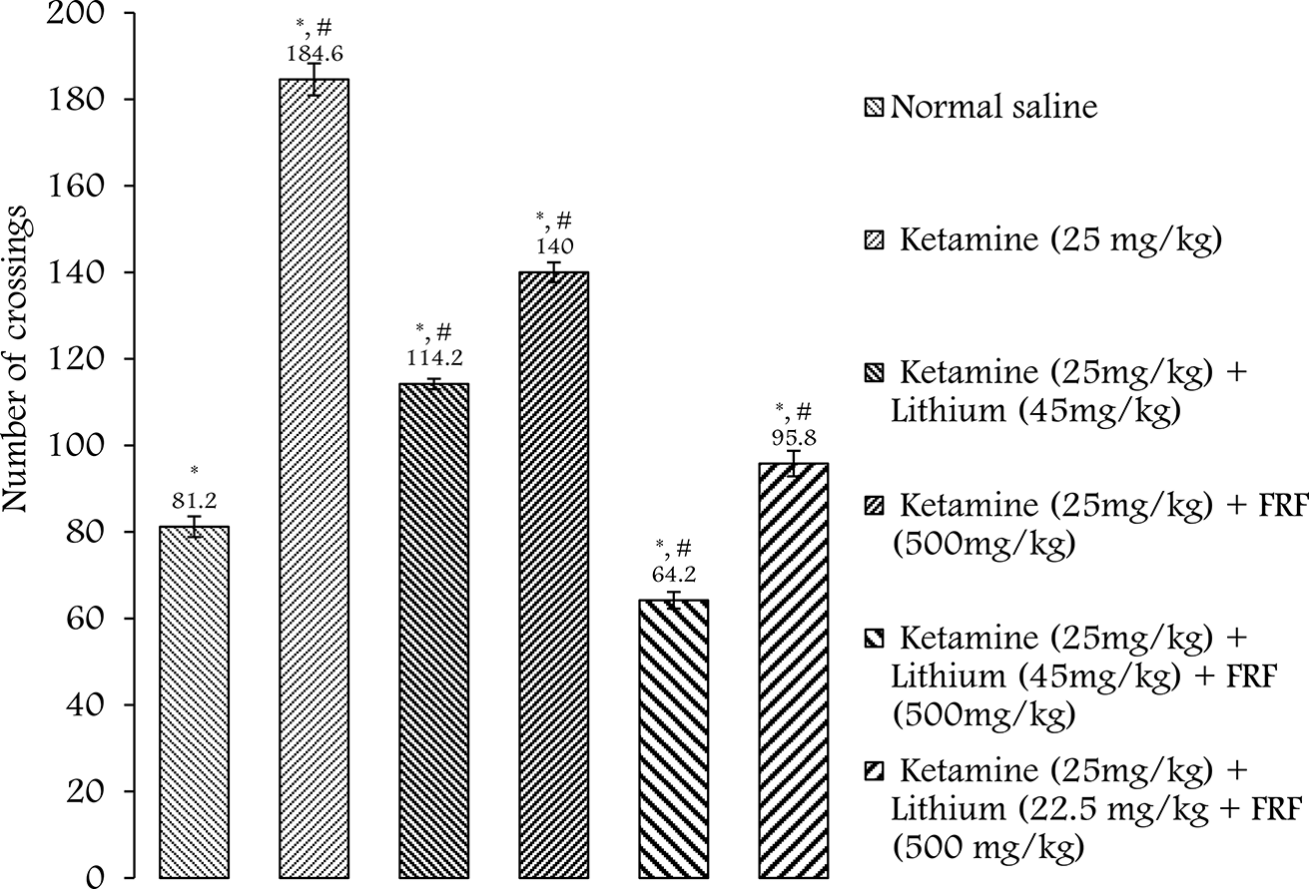
Effect of FRF of onion skin extract and Lithium chloride on ketamine-induced hyperactivity in the open-field locomotor activity test. The number of crossings was recorded. * Denotes *p* < 0.05 as compared to the control/normal saline group. # Denotes *p* < 0.00001 as compared to the control/ketamine group. Data were represented as means ± S.E.M. (One-way ANOVA, Bonferroni *post hoc* test)

A one-way ANOVA was performed to compare the effect of ketamine and/or FRF of Onion husk extract on ketamine-induced mania in rats. It revealed that there was a significant difference in mean hyperlocomotion-induced mania between at least some of the groups [F(6,30) = 204.40. *p* = 0.00]. As presented in Fig. 2., the Bonferroni posthoc analysis showed that treatment with ketamine was efficient in inducing hyperlocomotion in rats (*p* < 0.05), increasing the number of crossing in the open field test, which is indicative of mania-like behaviour in the animal model of mania. In an open field test, an increased number of crossings by an animal indicates heightened activity and reduced fear or anxiety towards open spaces, which is considered a mania-like behaviour. This behaviour is often associated with hyperactivity and impulsivity, resembling manic episodes observed in certain psychiatric disorders (Smith et al. 2016). Additionally, the FRF (500 mg/kg) and Lithium Chloride (45 mg/kg, used as positive control) pre-treatment prevented hyperlocomotion in the open-field test (*p* = 0.00 and *p* = 0.00).

### Effect of FRF of red onion (*Allium cepa* L.) husk extract on *neurochemical parameters*

#### Neurochemical parameters in the cerebral cortex

**Fig. 3:**
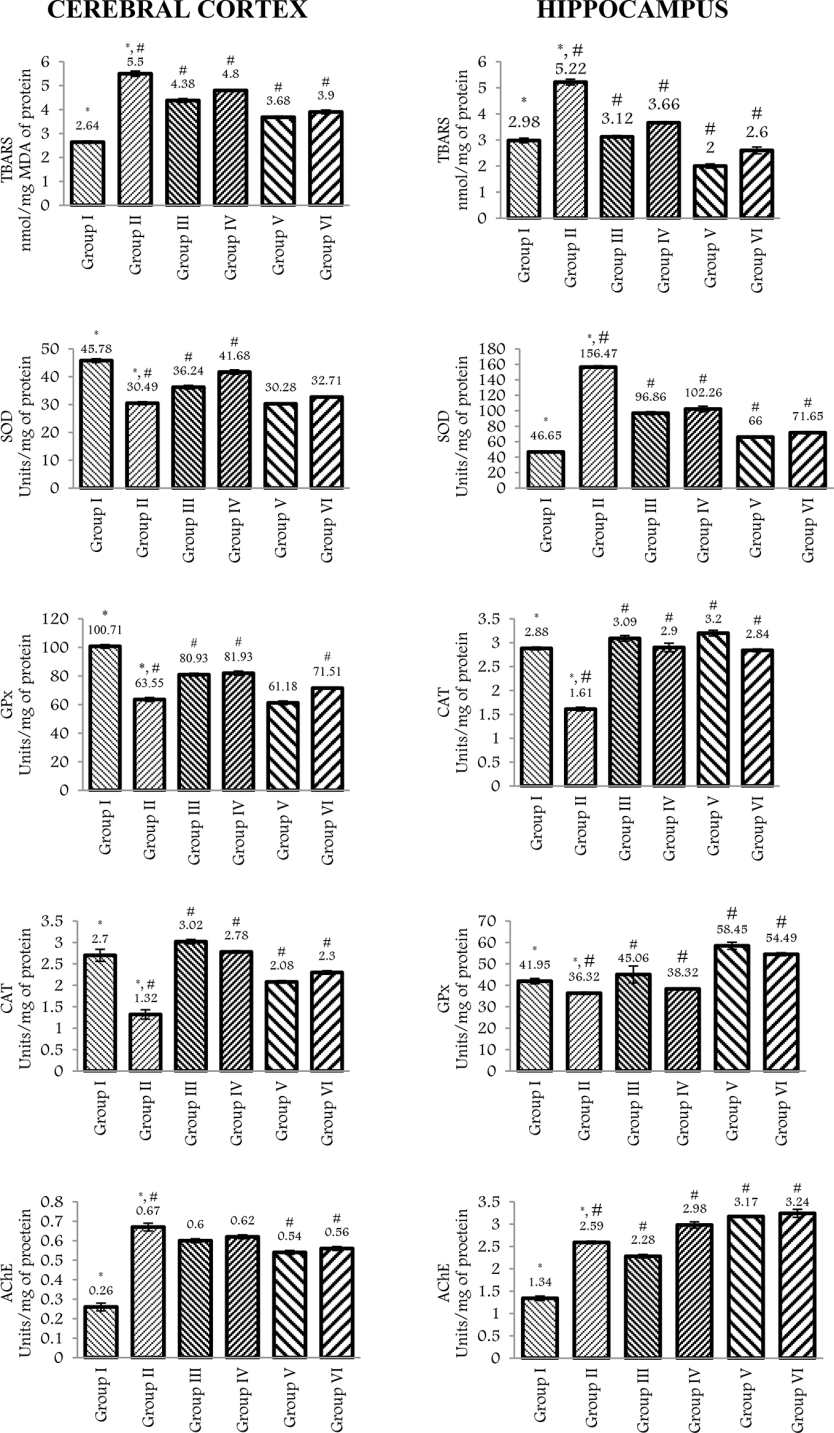
TBARS, SOD, GPx, CAT, and AChE in the cerebral cortex and hippocampus after pre-treatment (*n* = 5 for each group). Bars represent the standard error of the mean. *Denotes *p* < 0,001 compared to the normal saline group (Group I). #Denotes *p* < 0.001 as compared to the ketamine group (Group II). Group I: (Normal saline, vehicle); Group II: (Ketamine, 25 mg/kg); Group III: (Ketamine 25 mg/kg + Lithium 45 mg/kg); Group IV: (Ketamine 25 mg/kg + FRF 500 mg/kg); Group V: (Ketamine 25 mg/kg + Lithium 45 mg/kg + FRF 500 mg/kg); Group VI: (Ketamine 25 mg/kg + Lithium 22.5 mg/kg + FRF 500 mg/kg)

In investigating the biological underpinnings of mania-like behaviours, researchers can glean valuable insights by examining the neurochemistry of specific brain regions in rats. The *cerebral* cortex, responsible for impulse control and decision-making, is a prime target. Here, analyzing neurotransmitter levels might shed light on how chemical imbalances contribute to the impulsivity and poor judgment characteristic of mania. Ketamine administration (Group II) significantly increased TBARS levels compared to the control group (Group I) [F(1,8) = 524.33, *p* < 0.001], indicating elevated oxidative stress in the cerebral cortex. Lithium + FRF combination (Group V) significantly mitigated ketamine-induced TBARS elevation [F(1,8) = 201.97, *p* < 0.001], demonstrating its antioxidant potential. FRF alone (Group IV) also partially reduced TBARS levels [F(1,8) = 32.66, *p* < 0.01], suggesting some antioxidant effects. Interestingly, lithium alone (Group III) did not significantly affect ketamine-induced TBARS elevation compared to Group II. This suggests that FRF might play a more prominent role in the antioxidant effects of the combination. Similar to TBARS, ketamine (Group II) significantly decreased SOD activity compared to the control group (Group I) [F(1,8) = 304.94, *p* < 0.001], suggesting impaired antioxidant defence. Both FRF (Group IV) and lithium + FRF (Group V) pre-treatment partially restored SOD activity [F(1,8) = 135.58, *p* < 0.01 for Group IV; F(1,8) = 0.12, *p* = 0.728 for Group V]. This suggests that they might improve antioxidant defence capacity to some extent. Notably, the effect of lithium + FRF on SOD activity was not statistically significant compared to ketamine alone. This further supports the notion that FRF might be the main driver of the observed antioxidant effects. Ketamine (Group II) significantly downregulated GPx activity compared to the control group (Group I) [F(1,8) = 396.59, *p* < 0.001], indicating a reduced ability to remove harmful lipid peroxides. Similar to SOD, both FRF (Group IV) and lithium + FRF (Group V) pre-treatment partially restored GPx activity [F(1,8) = 80.96, *p* < 0.01 for Group IV; F(1,8) = 1.544, *p* = 0.2491 for Group V]. However, the effect of lithium + FRF was not statistically significant compared to ketamine alone. This again suggests that FRF might be the primary contributor to the observed improvement in GPx activity, with lithium potentially playing a less prominent role. Unlike other antioxidant enzymes, ketamine (Group II) significantly increased CAT activity compared to the control group (Group I) [F(1,8) = 57.01, *p* < 0.001]. This might indicate a compensatory mechanism in response to ketamine-induced oxidative stress. Interestingly, both FRF (Group IV) and lithium + FRF (Group V) pre-treatment significantly increased CAT activity compared to ketamine alone [F(1,8) = 154.46, *p* < 0.001 for Group IV; F(1,8) = 41.85, *p* = 0.728 for Group V]. This suggests that they might further enhance this potential compensatory mechanism. Similar to SOD and GPx, the effect of lithium + FRF on CAT activity was not statistically significant compared to ketamine alone. This reinforces the suggestion that FRF might be the main driver of the observed changes in CAT activity. Ketamine (Group II) significantly upregulated AchE activity compared to the control group (Group I) [F(1,8) = 148.41, *p* < 0.001], implying enhanced neuronal excitability. Lithium + FRF combination (Group V) significantly counteracted ketamine-induced AchE upregulation [F(1,8) = 28.32, *p* = 0.0007], offering neuroprotection against heightened neuronal excitability. Interestingly, neither FRF alone (Group IV) nor lithium alone (Group III) showed significant effects on AchE activity compared to ketamine alone (Fig. 3).

#### Neurochemical parameters in the hippocampus

The hippocampus, another key region of interest, plays a crucial role in mood regulation. By analyzing neurochemical changes within the hippocampus, researchers can investigate how these alterations might influence emotional processing and contribute to the mood swings experienced in mania. Ketamine administration significantly increased TBARS levels [F(1,8) = 230.16, *p* < 0.001] compared to controls, indicating elevated oxidative stress. SOD activity was concurrently decreased by ketamine [F(1,8) = 3584.17, *p* < 0.001], suggesting impaired antioxidant defence. Similarly, GPx activity was downregulated by ketamine [F(1,8) = 16.72, *p* = 0.003], further compromising antioxidant capacity. CAT activity also declined significantly in response to ketamine [F(1,8) = 428.79, *p* < 0.001], pointing towards reduced enzymatic antioxidant defence. Ketamine significantly upregulated AchE activity [F(1,8) = 326.12, *p* < 0.001], implying enhanced neuronal excitability. Pre-treatment with FRF mitigated ketamine-induced TBARS elevation [F(1,8) = 32.66, *p* < 0.01], demonstrating its antioxidant potential. SOD activity was also partially restored by FRF pre-treatment [F(1,8) = 135.58, *p* < 0.01], suggesting improved antioxidant defence. GPx activity showed a similar trend of partial recovery with FRF pre-treatment [F(1,8) = 80.96, *p* < 0.01]. CAT activity was significantly increased by FRF pre-treatment [F(1,8) = 154.46, *p* < 0.01], indicating enhanced enzymatic antioxidant capacity. Notably, FRF pre-treatment significantly counteracted ketamine-induced AchE upregulation [F(1,8) = 3.51, *p* < 0.01], offering neuroprotection against heightened neuronal excitability. Similar to FRF alone, pre-treatment with lithium + FRF alleviated ketamine-induced TBARS elevation [F(1,8) = 201.97, *p* < 0.01]. Additionally, the combination significantly reduced ketamine-upregulated AchE activity [F(1,8) = 28.32, *p* < 0.01], offering neuroprotection against neuronal overexcitation. Interestingly, no significant changes were observed in SOD, GPx, or CAT activity following pre-treatment with lithium + FRF. This suggests a differential effect of this combination on specific aspects of antioxidant defence mechanisms (Fig. 3).

**Table 6:** Biochemical and haematological data of wistar rats orally administered with FRF of onion husk extract in a 14-day repeat dose toxicity study

Parameters	Vehicle	Dose of FRF of Onion husk extract (mg/kg b.wt)		
	Group 1 (Distilled water)	Group 2 (500 mg/kg b.wt.)	Group 3 (1000 mg/kg b.wt.)	Group 4 (2000 mg/kg b.wt.)
Urea (mg/dl)	25.67 ± 1.37	27.59 ± 1.56	22.37 ± 2.23	27.85 ± 3.73
Creatinine (mg/dl)	2.16 ± 0.63	1.04 ± 0.05	1.50 ± 0.51	0.49 ± 0.14
Total protein (mg/dl)	5.93 ± 0.13^a, b^	9.68 ± 0.96^a, e^	10.76 ± 0.55^b, f^	5.44 ± 0.12^e, f^
Albumin (g/dl)	4.19 ± 0.38	3.70 ± 0.20	3.94 ± 0.07	3.88 ± 0.02
Globulin (g/dl)	1.74 ± 0.48^a, b^	6.01 ± 0.96^a, e^	6.84 ± 0.54^b, f^	1.56 ± 0.10^e, f^
Sodium ion (meq/l)	170.70 ± 2.63	183.91 ± 28.66	164.74 ± 15.85	184.84 ± 10.74
Bicarbonate (mmol/l)	13.18 ± 5.62	13.70 ± 2.25^e^	14.32 ± 0.98^f^	24.11 ± 1.25^e, f^
Chloride ion (meg/l)	56.59 ± 4.69	62.40 ± 2.47	59.04 ± 0.93	56.82 ± 1.76
Total bilirubin (µmol/L)	21.77 ± 2.68	18.28 ± 0.96	23.93 ± 2.44	19.51 ± 0.70
Conjugated bilirubin (µmol/L)	9.47 ± 0.61	8.80 ± 0.72	11.87 ± 0.30	8.58 ± 0.26
Alkaline phosphatase (iu/l)	10.31 ± 0.36^a, c^	6.66 ± 1.20^a^	6.33 ± 1.76	4.61 ± 0.64^c^
Alanine transaminase (iu/l)	15.58 ± 2.44c	11.91 ± 2.42	14.33 ± 0.68^f^	8.00 ± 1.04^c, f^
Aspartate aminotransferase (iu/l)	127.78 ± 9.53^c^	119.6 ± 3.93	168.82 ± 7.14^f^	154.13 ± 10.56^c, f^
Total White blood cell (×10^3^/µL)	9.16 ± 2.97	10.80 ± 8.61	10.16 ± 2.14	9.60 ± 1.58
Neutrophils (%)	21.33 ± 2.40	19.33 ± 1.85	19.33 ± 2.66	21.66 ± 1.45
Lymphocytes (%)	76.0 ± 1.52	80.33 ± 2.18^d^	78.33 ± 2.96^d^	78.33 ± 1.45
Eosinophils (%)	1.0 ± 0.57	0.33 ± 0.33	0.00 ± 00	0.33 ± 0.33
Monocytes (%)	0.00 ± 0.00	0.00 ± 0.00	0.00 ± 00	0.00 ± 0.00
Basophils (%)	0.00 ± 0.00	0.00 ± 0.00	0.00 ± 00	0.00 ± 0.00
Band Neutrophils (%)	1.66 ± 0.88	0.00 ± 0.00	2.33 ± 0.88	0.00 ± 0.00
Red blood count (×10^6^/µL)	4.95 ± 0.03	5.15 ± 0.15	5.21 ± 0.11	5.12 ± 0.11
Haemoglobin (g/L)	14.2 ± 0.15	15.16 ± 0.69	15.33 ± 0.35	15.33 ± 0.21
Mean Corpuscular Volume (µm^3^)	79.46 ± 0.58^b, c^	84.60 ± 1.17	85.66 ± 0.86^b^	84.60 ± 0.65^c^
Mean Corpuscular Haemoglobin (pg)	28.7 ± 0.11	29.4 ± 0.50	29.43 ± 0.26	29.93 ± 0.28
Mean Corpuscular haemoglobin concentration (g/dL)	36.10 ± 0.32	34.73 ± 0.14	34.36 ± 0.47	35.43 ± 0.12
Platelets (×10^3^/µL)	1.93 ± 0.02^c^	3.63 ± 0.36	2.08 ± 0.08^f^	2.97 ± 0.04^c, f^
Packed Cell Volume (%)	39.33 ± 0.33	43.66 ± 1.85	44.66 ± 1.45	43.33 ± 0.66

Data are expressed as mean ± SEM. Mean values with the same superscript letters are significantly different at *p* < 0.05

## Discussion

BD is a severe illness characterized by mood episodes ranging from mania to depression. Current medications effectively manage symptoms but come with side effects. This study explores the potential of onion skin, a natural source of antioxidants and bioactive compounds, as a novel therapeutic option. Research suggests oxidative stress and glutamate system dysregulation contribute to bipolar disorder. Onion skin’s flavonoids, particularly quercetin and kaempferol, possess potent antioxidant and anti-inflammatory properties, potentially mitigating the oxidative stress and neuroinflammation associated with the condition. Additionally, preclinical studies using relevant model systems are needed to investigate the influence of onion skin on the glutamate system and its potential impact on bipolar disorder pathophysiology. While the findings are promising, well-designed clinical trials are crucial to determine the safety, efficacy, and optimal dosage of onion skin or its isolated bioactive compounds for bipolar disorder treatment.

Onion skin emerges as a promising source of bioactive compounds with antioxidant and anti-inflammatory properties (Singh et al. 2016; Manach et al. 2005). These properties align with potential benefits for bipolar disorder, a condition marked by oxidative stress and neuroinflammation. Notably, onion skin contains flavonoids like quercetin and kaempferol, known for their neuroprotective effects (Harborne and Williams 2000). Collectively, these diverse bioactive compounds suggest onion skin holds immense promise as a readily available source of natural health-promoting agents. However, unlocking its full therapeutic potential and establishing safe utilization dosages require further comprehensive research.

Fourier-transform infrared (FT-IR) spectroscopic analysis of onion skin extract reveals a complex repertoire of functional groups indicative of a plethora of organic compounds (Silverstein and Webster 2014; Stuart 2018). The prominent peak at 3254.0 cm-1 signifies the abundance of hydroxyl (O-H) groups commonly found in alcohols, phenols, and carboxylic acids (Coates 2006). A characteristic peak at 2120.9 cm-1 suggests the presence of alkynes or nitriles, potentially contributing to the extract’s bioactivity (Smith 2011; Barth 2007). Aromatic and non-aromatic C = C bonds are evidenced by a peak at 1602.8 cm-1 (Smith 2011; Pavia et al. 2015), while nitro compounds or aromatic C = C bonds further manifest at 1509.6 cm-1 (Larkin 2011; Colthup et al. 1990). The peak at 1446.2 cm-1 confirms the presence of alkanes, alkenes, or alkynes (Smith 2011; Bellamy 1958). Amines, renowned for their diverse biological roles, are identified by the 1379.1 cm-1 peak (Nyquist 1996; Nakamoto 1986). Peaks at 1252.4 cm-1 and 1200.2 cm-1 indicate the presence of alcohols, esters, ethers, and aromatic C-O bonds, respectively (Smith 2011; Lin-Vien et al. 1991). The peak at 1166.7 cm-1 confirms carbon-carbon bonds, and polysaccharides, crucial for plant cell structure and function, are identified by the peak at 1036.2 cm-1 (Movasaghi et al. 2008; Coates 2000). These findings collectively suggest that onion skin extract harbours a rich tapestry of bioactive compounds, potentially offering a myriad of health benefits.

Gas chromatography-mass spectrometry (GC-MS) analysis of the FRF extracted from onion husks revealed a promising array of 17 bioactive compounds (Cho et al. 2023). Five-methyl-2-phenylindolizine, constituting nearly half of the FRF, emerged as the most abundant compound (Cho et al. 2023). Methadone N-oxide, 3-phenylamine, S-oxide, and benzene, 1, 1’-(1, 2-cyclobutanediyl)bis-, trans- were identified as other major contributors (Cho et al. 2023). While the specific effects of these compounds require further investigation, some, like n-hexadecanoic acid and 5-eicosene, have shown potential antioxidant activity (Iqbal and Bhanger 2006; Kubo et al. 1994). Additionally, 3-phenylthiane, S-oxide has been reported elsewhere to demonstrate anti-inflammatory properties in preclinical studies and methadone N-oxide exhibits potential antimicrobial activity against specific bacteria (Sheagren et al. 1977). Notably, compounds like 5-methyl-2-phenylindolizine and (1-ethylbuta-1,3-dienyl)benzene have shown interactions with the nervous system, warranting further investigation into their specific effects (Li et al. 2007; Yagi et al. 2001).

The FRF of onion husk extract demonstrated excellent safety profiles in both single-dose and 14-day repeated-dose toxicity studies in rats. No significant changes were observed in body weight, organ weights (liver, kidney, heart), or haematological parameters, suggesting minimal impact on metabolism, energy balance, and overall health. The absence of organ damage in vital organs like the liver and kidneys further reinforces the low toxicity and potential safety of the FRF for consumption. These findings align with previous research on the non-toxic nature of onion husks and their extracts (Lee et al. 2021; Piechowiak and Balawejder 2019), and pave the way for further exploration of the FRF’s potential as a dietary supplement or functional food ingredient (Sharma et al. 2020).

While no adverse effects were observed at the tested doses (300 mg/kg and 2000 mg/kg) in the single-dose and 14-day repeated-dose studies, some minor fluctuations in blood parameters warrant further investigation. Increased band neutrophils in the FRF-treated groups may indicate early inflammation or stress response (Layton et al. 2023), necessitating further exploration of inflammatory markers. Elevated MCV and platelet fluctuations across groups also require additional monitoring to clarify potential influences on cell volume and bone marrow function, respectively (Aslinia et al. 2006). Notably, all other haematological parameters, including renal function markers (urea and creatinine), remained within normal ranges, implying minimal impact on major organ systems.

Histopathological assessments of the liver, kidney, and heart revealed no abnormalities in any of the FRF-treated groups, further corroborating the lack of organ damage and supporting the extract’s safety profile. These findings are consistent with the positive safety profile reported for *Allium cepa* peels by Builders et al. (2023) and the potential antioxidant effects observed in the liver and brain of rats by Chernukha et al. (2021).

Overall, the present study provides compelling evidence for the low toxicity and potential safety of the FRF of onion husk extract for oral consumption. However, future investigations with larger sample sizes and longer treatment durations are crucial to confirm the long-term safety and potentially identify any delayed-onset adverse effects (Berlin et al. 2008). Additionally, exploring the mechanisms of action and effects on other organ systems and physiological processes will further elucidate the full therapeutic potential of this promising natural extract.

Our findings demonstrate that FRF pretreatment significantly attenuates ketamine-induced hyperactivity in the open-field test, mirroring the effect of the established mood stabilizer lithium chloride (Debom et al. 2016). Additionally, FRF treatment reduces acetylcholinesterase (AChE) activity in these brain regions, indicating modulation of the cholinergic system (Haam and Yakel 2017). The observed antioxidant properties of FRF are attributed to the presence of n-hexadecanoic acid and 5-eicosene, known for their potent antioxidant activity exceeding α-tocopherol and catechin (Iqbal and Bhanger 2006; Kubo et al. 1994). These findings align with previous studies demonstrating the efficacy of plant extracts rich in phenolic compounds in mitigating ketamine-induced hyperlocomotion (Debom et al. 2016; Gazal et al. 2015; Chaves et al. 2020). While the ketamine model lacks perfect specificity for mania (Sharma et al. 2016), the observed effects of FRF in preventing hyperlocomotion, reducing oxidative stress, and modulating cholinergic activity provide compelling evidence for its potential as a therapeutic candidate for BD. Future research using larger sample sizes and diverse models is necessary to validate these findings and elucidate the exact mechanisms of action underlying FRF’s neuroprotective effects. This suggests the potential utility of FRF in managing manic episodes associated with BD. Mechanistically, FRF appears to counteract ketamine’s effects by mitigating oxidative stress markers like elevated TBARS and ROS levels in the cerebral cortex and hippocampus (de Oliveira et al. 2009).

This research opens promising avenues for exploring the potential of FRF as a natural, potentially less-invasive therapeutic option for BD management. Further investigation into FRF’s efficacy and safety profile alongside its interaction with established BD medications is crucial before clinical translation.

## Conclusion

Onion husks, a waste product rich in antioxidants and potentially beneficial for health, were investigated in this study for their effect on manic behaviour in rats. The extract proved effective, highlighting the therapeutic value of the food waste products. While the present study provides compelling evidence for the low toxicity and potential safety of the onion husk extract for oral consumption, further investigations with larger sample sizes and longer durations are crucial to confirm long-term safety and identify any delayed adverse effects. Additionally, exploring the mechanisms of action and effects on other organ systems and physiological processes will further elucidate the full potential of this promising natural extract.

## Data Availability

The data that support the findings of this study are available from the corresponding author upon reasonable request.
